# Biomining of metals: how to access and exploit natural resource sustainably

**DOI:** 10.1111/1751-7915.12792

**Published:** 2017-08-03

**Authors:** Carlos A. Jerez

**Affiliations:** ^1^ Laboratory of Molecular Microbiology and Biotechnology Department of Biology Faculty of Sciences University of Chile Santiago Chile

## Abstract

Mining activities have been carried out for thousands of years and nowadays have an enormous worldwide use to obtain important metals of industrial use. These include copper, iron, gold and several others. Although modern mining companies have sustainable mining programs that include tailings management and external verifications, it is recognized that these industrial activities are responsible for a significant damage to the environment. Specially, technologies such as smelting and roasting generate very toxic emissions, including solid particles in the air, very large tailings and contribute to generate acid mine drainage (AMD) that affects humans health and all kinds of living plants, animals and microorganisms. Consequently, due to environmental restrictions, these methods are being replaced in many countries by less contaminating processes. On the other hand, the microbial solubilization of metals by bioleaching or biomining is successfully used in industrial operations, to extract several metals such as copper, gold and uranium.

## Abstract 2

How to access and exploit natural resources sustainably by using biomining of metals.

## Mining of metals

Mining activities have been carried out for thousands of years and nowadays have an enormous worldwide use to obtain important metals of industrial use. These include copper, iron, gold and several others. Although modern mining companies have sustainable mining programmes that include tailings management and external verifications, it is recognized that these industrial activities are responsible for a significant damage to the environment. Specially, technologies such as smelting and roasting generate very toxic emissions, including solid particles in the air, very large tailings, and contribute to generate acid mine drainage (AMD) that affects human health and all kinds of living plants, animals and microorganisms. Consequently, due to environmental restrictions, these methods are being replaced in many countries by less contaminating processes. As accessible primary metal ores become lower grade, the recycling of mine wastes becomes more viable and economically appealing. For example, substantial re‐processing of tailing dumps and deposits is currently under way in South Africa. This in turn reduces the problem of AMD (Harrison, 2016).

On the other hand, the microbial solubilization of metals by bioleaching or biomining is successfully used in industrial operations, to extract several metals such as copper, gold and uranium. It is commercially applied using dumps, heaps and stirred tanks. This is an important biotechnological procedure used in many countries that generates several hundred thousand tons a year of metals such as copper.

A consortium of different iron‐ or reduced inorganic sulfur compounds (RISCs)‐oxidizing microorganisms participates in the oxidative reactions of minerals, resulting in the extraction of dissolved metal values from ores and the generation of ferrous iron and sulfate. The microbes oxidize ferrous iron to ferric iron that is a strong oxidant capable to further oxidize metal sulfides. This greatly accelerates the mineral dissolution rate. As chalcopyrite is one of the most abundant and difficult minerals to solubilize by microorganisms, there is actually great interest in developing processes using thermophilic biomining microorganisms for these recalcitrant metal sulfides. The extraction of copper from chalcopyrite using bioreactors can be faster and efficient at temperatures higher than 60°C using thermophilic archaea. These operations are easier to control compared with standard bioleaching using ore heaps (Martínez‐Bussenius *et al*., 2017).

Although biomining offers an economically viable and cleaner option, the acidophilic microorganisms, mobilize metals and also generate AMD therefore still causing environmental harm.

## Bioremediation of metal‐contaminated areas

The best situation for mining activities would be to avoid the generation of the highly toxic secondary products such as acid and non‐recovered metals. If this is not likely, AMD should be remediated or abated. Often there is a sealing of the contaminated sites or barriers are located to contain the acidic fluids (Klein *et al*., 2014). Many approaches use prevention techniques to circumvent further spillage of acidic effluents in the polluted area. It can be controlled by chemical treatments such as the use of calcium oxide that neutralizes the acid pH. It is also possible to inhibit the acidophilic microorganisms responsible of the acid generation. This can be done using certain organic acids, sodium benzoate, sodium lauryl sulfate or quaternary ammonium compounds that will affect the growth of bacteria such as *Acidithiobacillus ferrooxidans*. Many of these treatments are complicated to apply and are expensive.

Bioremediation or removal of the toxic metals from contaminated soils can be achieved using the specific properties of microorganisms interacting with metals. Knowing how acidophiles survive in acid and metal‐rich locations is important to have a better approach for biomining of metals and also for bioremediation of AMD places and most likely to improve and generate new biotechnological cleaning processes.

A very interesting example of toxic metals elimination from contaminated soils has been the use of a combination of two opposite biological activities: that of sulfur‐oxidizing bacteria with the one of sulfate‐reducing microorganisms. In a first step, the sulfur‐oxidizing bacteria generate sulfuric acid which bioleaches or solubilizes the metals in the solid phase of the soil. The leachate metals are then precipitated in a second step using a bioreactor in which the hydrogen sulfide generated by the sulfate‐reducing bacteria under neutral and anaerobic conditions forms insoluble metal sulfides, which can be contained or used to recover the precipitated metals. Metal contaminants such as Cu, Cd, Ni and others can be efficiently leached from filthy soils. The effluents obtained from such a process are clean enough of the metals that they can be reused in the environment.

Bioleaching also has the potential for ex situ on site low‐cost remediation of aquatic sediments contaminated with metals. Improvements of these procedures will still require to understand further the geochemical characteristics of the sediment, the partitioning of the contaminating metals and the biological mechanisms involved (Fonti *et al*., 2016).

## The use of microorganisms to recover special metals from waste can avoid environmental pollution

Bioleaching microorganisms such as *A. ferrooxidans* can also have other uses to help avoiding metal contaminations in modern societies. For example, this and other bacteria have been successfully used to recuperate metals such as cadmium from spent batteries. Using bioreactors, *A. ferrooxidans* is grown attached on elemental sulfur. Bacteria generate sulfuric acid through the oxidation of sulfur that is then used for the indirect dissolution of spent nickel–cadmium batteries recovering 90%–100% of cadmium, nickel and iron after 3 months. Bioleaching of spent lithium ion secondary batteries, containing lithium and cobalt, has also been explored. These approaches are not only economically valuable but may be an effective method which could be considered the first step to recycle spent and discarded batteries preventing one of the many problems of environmental pollution (Zhuang *et al*., 2015). In addition, urban wastes such as e‐wastes are being used to recycle or recover metals because some of these discarded wastes have richer metal grades than ores, as in the case of copper.

The critical increasing necessity of platinum group metals and rare earth elements such as terbium, europium and others especially due to the augmented development of green low‐carbon energy technologies that require these metals has been highlighted (Zhuang *et al*., 2015). For this, biometallurgy is advantageous, being specific, requiring low energy and creating minimal new waste.

## Future improvements of biomining

Recent OMICs (genomics, proteomics, transcriptomics, metabolomics) advances have greatly helped the study of individual members of the biomining microbial community to understand better the mechanisms used to grow and adapt to their harsh environment. The great facilities existing these days for genome sequencing is allowing to study the entire consortium microbiome. Although some procedures to genetically modify a few of the biomining microorganisms are available, greater advances are required in this area (Jerez, 2017). This in turn would allow to genetically modify the entire extremophilic microbiome to obtain much more efficient and controlled metal concentration processes, redefining the current metal extraction sequence (Dunbar, 2017). Developments of ‘laboratory evolution’ mixtures to find higher metal tolerances, improved attachment to minerals, growth at higher minerals pulp concentrations and faster growth can be envisaged in the future.

## Space biomining?

Amongst the grand challenges for space synthetic biology, enhancing biomining and bioleaching for asteroid and planetary deployment has been indicated as one of many important synthetic biology possibilities (Menezes *et al*., 2015). Recently, the possibility to bioremediate mining wastewaters using microbial fuel cell technology with acidophilic microorganisms has been explored (Ni *et al*., 2016). Fuel cells can be generated to discard many other inorganic and organic wastes, and the electricity generated is an extra bonus. Obviously, the idea in this case should be to use these and other similar advances not only to avoid the great contaminations that have been generated by humans to obtain metals in planet Earth but also to have a totally sustainable microbial ‘planetary mining’.

## Conflict of interest

None declared.
